# Combating Burnout Amongst Residents Through Fostering Resilience: A Systematic Review

**DOI:** 10.1177/10499091251331150

**Published:** 2025-03-28

**Authors:** Le Xuan Shannon Tan, Jiayan Ding, Timothy Xuxian Neo, Vincent Sixian Chen, Charmaine Shi Mei Lee, Nur Amira Binte Abdul Hamid, Yun Ting Ong, Lalit Kumar Radha Krishna

**Affiliations:** 1 63751 Yong Loo Lin School of Medicine, National University of Singapore, Singapore; 2 Division of Cancer Education, 68751 National Cancer Centre Singapore, Singapore; 3 Division of Supportive and Palliative Care, 68751 National Cancer Centre Singapore, Singapore; 4 Palliative Care Institute Liverpool, Academic Palliative & End of Life Care Centre, Cancer Research Centre, 4591 University of Liverpool, Liverpool, UK; 5 Duke-NUS Medical School, National University of Singapore, Singapore; 6 Centre for Biomedical Ethics, National University of Singapore, Singapore; 7 Health Data Science, 4591 University of Liverpool, Liverpool, UK; 8 PalC, The Palliative Care Centre for Excellence in Research and Education, Singapore

**Keywords:** resilience, burnout, grit, residents, personhood, compassion fatigue, costs of caring

## Abstract

**Background:**

Burnout, marked by emotional exhaustion, depersonalization and depletion of professional efficacy, is rampant among residents. With deleterious effects on productivity, conduct and patient care, resident programs have increasingly emphasized resilience training to combat burnout. However, the personalized nature of burnout complicates the effective design of such programs. Premised on recent works that identify burnout as a result of shifting personhood, this study utilizes the Ring Theory of Personhood to guide the conceptualization of resilience training programs that address changes in the belief systems shaping one’s self-concept.

**Methods:**

A systematic scoping review to explore how resilience is addressed and assessed amongst medical residents was conducted. Guided by the PRISMA-compliant Systematic Evidence-Based Approach (SEBA), searches for relevant articles published between 1st January 2000 and 4th November 2024 on PubMed, Embase, Scopus, ERIC and PsycINFO were performed. The SEBA methodology facilitated the integration of the themes and categories identified using thematic and content analyses.

**Results:**

Of 15 953 abstracts screened, 666 articles were reviewed and 69 full-text articles were included. Three domains were identified: guiding theories; methods of teaching resilience; and assessment.

**Conclusion:**

The reliance on individual or societal theories has constrained the understanding, design and assessment of resilience programs. Current approaches, including mindfulness workshops, self-care initiatives and organization-led resilience training, are neither timely nor focused on the needs of each resident. To mitigate burnout, personalized, longitudinal and timely support is essential. Mentoring offers a more suitable alternative, providing culturally sensitive, resource-appropriate, sustainable and clinically relevant support to build resilience effectively.

## Introduction

With burnout rates amongst residents as high as 50% across the globe, greater focus has been placed on this psychological syndrome characterized by emotional exhaustion, depersonalization and a reduced sense of personal accomplishment or professional efficacy.^1-12^ These concerns are far-reaching, with ramifications on the wellbeing, productivity, conduct and empathy of health care professionals—impacting patient safety and care.^1,6,8,9,11-34^

Influenced by the notion that burnout is predisposed by an inability to cope, adapt and recover from adverse situations, many resident programs have sought to instill resilience—the capacity to recover following a setback—amongst its residents as part of wider efforts to enhance working conditions, rewards and recognition, alongside improving training and working culture.^2,4,7-10,12,21,23,31,34-43^ Yet, whilst there have been recent reviews on the subject, there has not been significant headway made on how to design effective resilience training programs for residents, partly due to the personalized approach needed.

McKinley et al,^24^ Howard et al^44^ and Seo et al^3^ highlight the personalized nature of burnout. Recognition of sociocultural influences and population differences may explain the diversity in approaches to teaching resilience identified in current reviews.^3,26,45-48^ Dyrbye and Shanafelt^49^ and Angelopoulou and Panagopoulou’s^50^ suggest that a multipronged approach pivoting on Emotional–Supportive-Coping interventions is required whilst Howard et al^44^ recommend the use of more holistic assessments.

Based on recent work that identifies burnout and compassion fatigue as the products of shifts in an individual’s sense of personhood (the notion of ‘what makes you, you’), this systematic scoping review aims to map prevailing literature on burnout and resilience training, with the end goal of guiding the conceptualization of effective resilience training programs using the clinically evidenced Ring Theory of Personhood.^51-55^ It is proposed that personalized and timely support of the belief systems embedded in one’s self-concept of personhood is more effective in addressing the effects of burnout.

### The Ring Theory of Personhood

**Figure 1.**
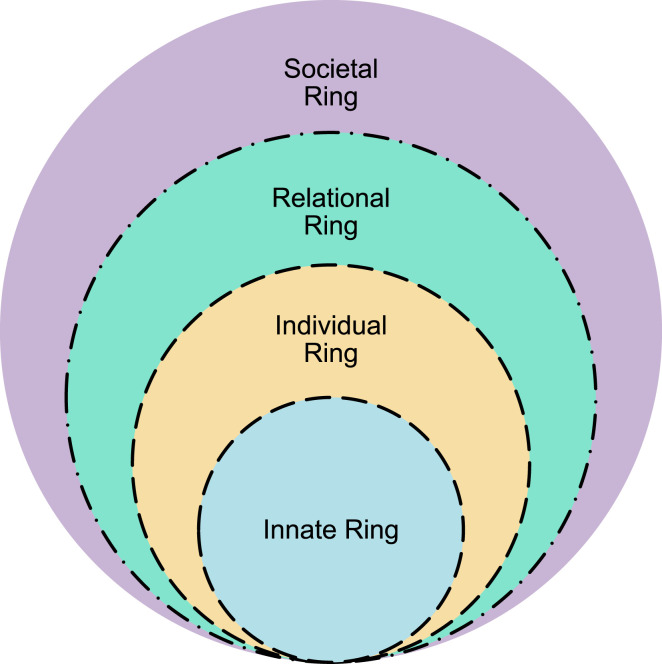
The ring theory of personhood.

The Ring Theory of Personhood (RToP) conceptualizes personhood as four intertwined rings, namely the Innate, Individual, Relational and Societal Rings (Figure 1).^56-59^

Each ring houses a unique set of belief systems that correspond to different domains of personhood. The Innate Ring includes belief systems tied to the physician’s demographic features and spiritual and/or religious beliefs. The belief systems in Individual Ring pertain to the physician’s conscious function and personality, which may manifest as thoughts, behavior and speech. The Relational Ring encompasses belief systems surrounding close personal relationships, such as family and close friends, whilst the Societal Ring pivots on belief systems that inform sociocultural, professional, legal and ethical norms, expectations, rights, roles and responsibilities. Changing belief systems brought on by new experiences influence a physician’s self-concept of personhood.

## Methods

**Figure 2.**
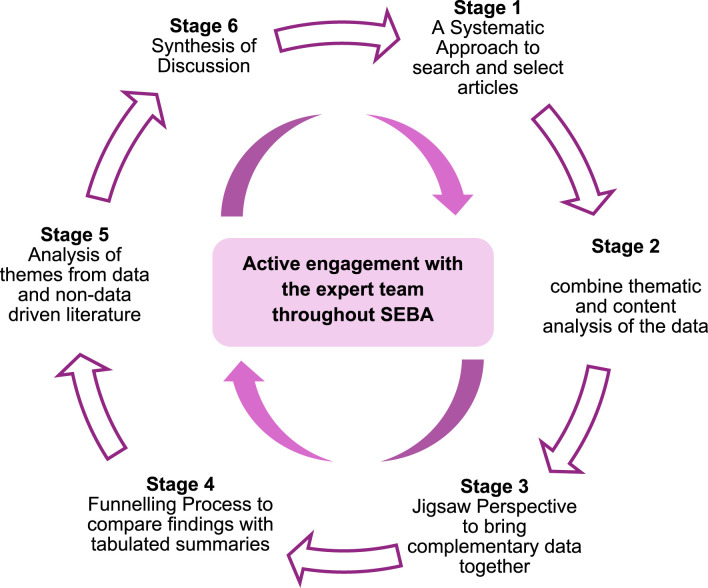
Stages of the systematic evidence-based approach.^71^

To structure this review, the six-staged Systematic Evidenced-Based Approach (SEBA) (Figure 2) was adopted as the methodological framework.^60-63^ Its positivist, constructivist ontological and relativist epistemological approach is apt in instilling consistency, reproducibility and trustworthiness to accommodate various personal and sociocultural factors and practice settings whilst complying with standard PRISMA guidelines (Additional File 1).^64-70^ Further enhancing the reliability and transparency of this process was the expert team of medical librarians, local educational experts and clinicians guiding the research team in all stages of SEBA.

### Stage 1 of SEBA: The Systematic Approach

#### Determining the Research Question and Inclusion Criteria

Guided by clear inclusion and exclusion criteria aligned with the Population, Context, Concept (PCC) framework (Table 1), this review focused on addressing our primary research question: *What is known about how resilience is addressed amongst medical residents?* The secondary research questions included: *What approach and content are used to build resilience amongst medical residents? How is resilience assessed amongst medical residents?*

**Table 1.** Inclusion and Exclusion Criteria Applied to Database Search.

	Inclusion Criteria	Exclusion Criteria
Population	Physicians who are undergoing residency training in any clinical specialty, in any country	Medical students, physicians not in residency or clinical practice
		Allied health specialties
		Clinical and translational science, veterinary, dentistry
Context	Full text articles in English or translated to English	Grey literature
	All study designs: Mixed methods research, meta-analyses, systematic reviews, randomized controlled trials, cohort studies, case-control studies, cross-sectional studies, descriptive papers	
	Year: 1^st^ Jan 2000 – 4^th^ Nov 2024	
Concept	Articles discussing the nurturing of resilience in the context of physician burnout	

#### Searching

Between 4^th^ June 2023 and 4^th^ November 2024, the research team conducted searches on PubMed, Embase, Scopus, ERIC and PsycINFO databases for articles published from 1^st^ January 2000 to 4^th^ November 2024. Additional File 2 details the search strategies applied to the database search.

#### Extracting and Charting Data

Utilizing EndNote for abstract screening, two pairs of research team members independently reviewed abstracts shortlisted by the search strategy. If both members agreed that an abstract failed to meet the inclusion criteria, it was excluded from further independent full-text review. Discrepancies were resolved through discussion and consensus, with a third member acting as a tiebreaker when necessary.

The final list of articles included for full-text reviews was subsequently summarized using ChatGPT 4o (see Additional File 3).

### Stage 2 of SEBA: Split Approach

The included full-text articles subsequently underwent concurrent thematic and content analyses. This combined method of data analysis facilitated a more comprehensive and nuanced analysis that saw the limitations of each method offset by the other. For instance, the subjective nature of thematic analysis is balanced by the objectivity in content analysis while the lack of depth in content analysis is accounted for by thematic analysis.

#### Thematic Analysis

The first team of researchers performed Braun and Clarke’s^72^ approach to thematic analysis to identify meaningful patterns in the data. The surface meanings of these patterns were then extracted to form codes. In an iterative step-by-step analysis process, each emerging code was linked to previous complementary ones that, together, led to the synthesis of themes.^73^ Through discussion and negotiation within the team, consensus on the key themes was reached.^74^ Inter-rater reliability was not evaluated as the teams held regular meetings to discuss and compare their findings following their reviews of a specified number of similar articles.

#### Directed Content Analysis

Concurrently, the second research team applied Hsieh and Shannon’s^75^ directed content analysis using pre-existing codes drawn from Sanjaya et al.’s^76^ study entitled ‘*Resilience: A panacea for burnout in medical students during clinical training? A narrative review*’. Texts of similar meaning were grouped into categories whilst new codes were assigned to any data uncaptured by pre-existing ones. Agreement on the key categories was similarly achieved through team discussions.

### Stage 3 of SEBA: The Jigsaw Perspective

The Jigsaw Perspective maintains that complementary qualitative data bears *“a richer, more nuanced understanding of a given phenomenon”*.^77^ In this regard, the research team reviewed the identified themes and categories, with overlapping and/or complementary data combined to form broader units called themes/categories, akin to pieces of a jigsaw puzzle.

### Stage 4 of SEBA: The Funneling Process

To ensure that pivotal features of each article were retained, the research team compared the identified themes/categories against the tabulated summaries. This systematic comparison and juxtaposition of data formed key domains that underlined the ensuing discussion. Phases 3 to 6 of France et al.’s^78^ adaptation of Noblit and Hare’s^79^ seven phases of meta-ethnography were utilized to assist with this process.

### Stage 5 of SEBA: Analysis of Evidence-Based and Non-Data Driven Literature

The inclusion of non-peer-reviewed and non-evidence-based literature might have influenced the data. To assuage this concern, the research team compared the themes/categories from non-data-driven literature with that of evidence-based articles. With both groups yielding similar data, non-data-driven literature was unlikely to have biased the analysis.

### Reflexivity and Reiterative Process

The SEBA methodology emphasized the importance of reflexivity where researchers examined and considered how their individual beliefs, experiences and assumptions had influenced the research process. This prompted the research team to consistently reflect on emerging themes and categories, a process performed independently and collectively through regular discussions. Engaging in reflexivity safeguarded a more trustworthy and nuanced analysis that accommodated diverse personal and sociocultural factors.

Moreover, as part of SEBA’s reiterative process, the research team continuously reviewed new data that emerged in the themes, categories and domains formed. Engaging with the expert team in the same manner, with repeated consultations as new data emerged, further enhanced the analysis. This repeated review and comparisons of data added to more a robust and transparent data analysis.

## Results

**Figure 3.**
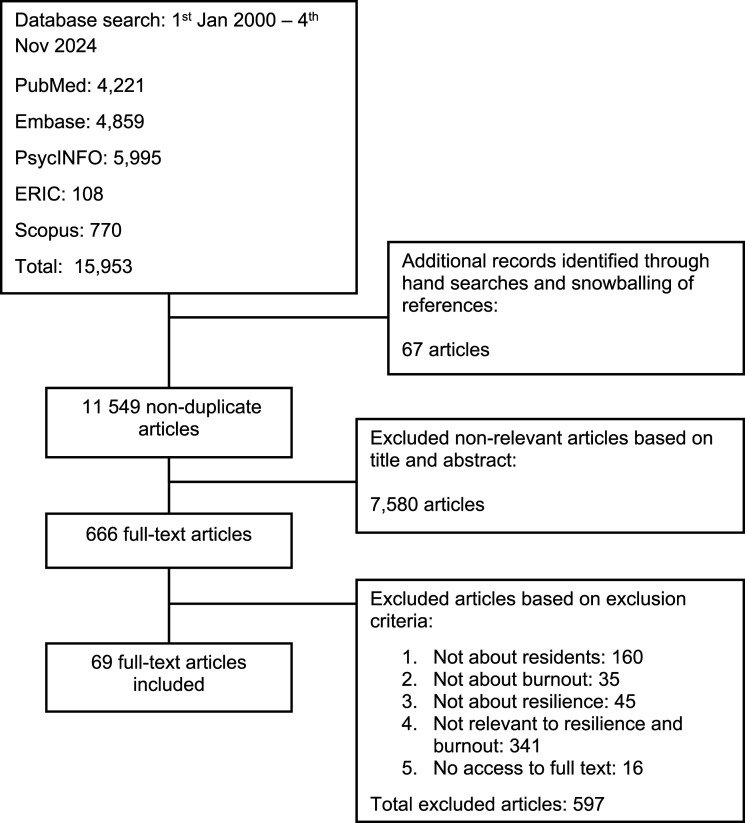
Prisma Flowchart.

A total of 15 953 abstracts were identified from five databases, of which only 666 papers were assessed to be eligible. 69 full-text articles were then included for this review (Figure 3). Three key domains were identified: 1) guiding theories; 2) methods of teaching resilience; and 3) assessment.

### Domain 1. Guiding Theories

The main theories guiding regnant understanding of resilience are summarized in Table 2. These theories examine how resilience interacts with influencing factors, such as burnout, organizational systems, social support and grit.

**Table 2.** Main Theories Guiding Understanding of Resilience.

Concepts Discussed	Total Articles	Single-Theory	Multi-Theory
Resilience and its relationship with burnout	45	25	20
Resilience (only without discussion of other concepts)	38	16	22
Resilience and its relationship with organizational systems	14	8	6
Resilience and its relationship with social support	12	6	6
Resilience and its relationship with grit	8	4	4

### Domain 2. Methods of Teaching Resilience

A variety of methods is employed to teach resilience. This can be viewed from the lens of the RToP. Interventions impacting the Societal Ring comprise curriculum reform, work-life balance, crisis management and digital resilience training. Those modulating the Individual Ring include mindfulness training, grit building, positive psychology, coping reservoir, resilience coaching and time management. Interventions directly impacting the Relational and Innate Rings are few, with those aimed at the Individual and Societal Rings often impacting these rings through spillover effect.

#### Individual Stress Management Techniques

Individual stress management techniques include mindfulness practices, such as meditation, breathing exercises and stress management skills.^21,23,27,34,37,40,80-82^ These methods empower residents and promote emotional regulation as a means of stress reduction.^37^

These programs begin with workshops that highlight the role of self-compassion, reframing and self-awareness.^4^ Such workshops also encourage realistic goal-setting, managing expectations and nurturing reflection and gratitude, with an added benefit of cultivating emotional intelligence and personal growth.^30,83^ Rodríguez-Socarrás et al^9^ note that the SWADDLE program from Baylor Scott and White Health; the LiveWell program of the Carolina health system; and the WellMD of the Stanford health systems exemplify this approach. Meanwhile, Bird et al^83^ introduce a resilience workshop that provides residents with a comprehensive understanding of resilience, accompanied by practical strategies to strengthen it. The workshop focuses on setting realistic expectations, processing and moving forward after stressful clinical experiences, alongside cultivating gratitude.

#### Self-care and Wellness

Self-care and wellness are essential to resilience.^20^ Encouraging good work-life balance, with dedicated time for hobbies, relaxation and quality time with friends and family, helps create a well-rounded lifestyle.^15,20,37,84^ This includes sleep hygiene, nutrition and regular physical activity to support mental and physical well-being.^23,37,85^

#### Structured Training and Support

Structured resilience training provides residents with essential skills and supportive resources to manage stress and build resilience. This includes cognitive-behavioral techniques and role-play exercises facilitated by close supervision, mentorship and buddy programs to boost interpersonal communications and manage emotions.^18,21,27,43,84,86-88^ McFarland and Roth^89^ propose group support to foster positive attitudes and confidence whilst Zoorob et al^88^ highlight access to educational support and institutional resources to strengthen resilience. Martinchek et al^4^ note that enabling residents to apply resilience concepts to practice is also pivotal.

#### Building Strong Social Support Network

Establishing strong social support networks is vital for enhancing resilience among residents.^24^ This was particularly evident during the early COVID-19 pandemic where social support networks, mentorship and personalized communication with leadership reduced stress and fostered teamwork, camaraderie and a sense of belonging among residents—building resilience.^84,88^

#### Organizational Initiatives

It is here that the role of the host organization and health care institution in nurturing resilience becomes evident. Structured facilitated discussion groups to create safe spaces for residents to express their feelings, share challenges and receive encouragement from peers; the cultivation of a working environment where residents can feel valued and understood; and the provision of meaningful professional support and relationships encourage resilience.^14,87,90^

Corgan et al^15^ and Franco et al^43^ further note that flexible scheduling and improved working hours alleviate stress, boost well-being, enhance efficiency and foster a culture of wellness that promote resilience.^21^

### Domain 3. Assessment

A variety of methods is utilized to assess resilience and burnout (Table 3).

**Table 3.** Tools to Assess Resilience and Burnout.

Concepts Discussed	Total Articles	Single-Theory	Multi-Theory	Tool and Number of Articles	Tool Focus	Tool Gap
Resilience and its relationship with burnout	45	25	20	MBI 52	Emotional exhaustion (EE) – Measures energy depletion	Fails to capture external systemic contributors, such as workload policies, staffing levels and leadership deficiencies
					Depersonalization (DP) – Assesses detachment and cynicism	
					Personal accomplishment (PA) – Evaluates feelings of inefficacy	
				CD-RISC 34	Personal resilience	
				Institutional metrics 15	Workplace conditions, scheduling practices and leadership quality	

Resilience (only without discussion of other concepts)	38	16	22	CD-RISC 34	Personal resilience	
				PPS 10	Current stress level	Snapshot
Resilience and its relationship with organizational systems	14	8	6	Institutional metrics 15	Workplace conditions, scheduling practices and leadership quality	

Resilience and its relationship with social support	12	6	6	Social support questionnaire 9	Perceived levels, quality and availability of peer, mentor and supervisor support	Self-reporting bias
				MBI 7		
				Qualitative interviews 7	Rich nuanced data	Time-sensitive
						Context-specific
Resilience and its relationship with grit	8	4	4	Grit scale 8	Psychological attributes	No environmental data
						Self-reporting bias
				CD-RISC 4		

These tools primarily consider the Societal and Individual Rings but rarely together. There were no tools that considered longitudinal effects of resilience and resilience training.

## Discussion

This review affirms the growing need for a more holistic view of resilience. Current conceptions of resilience as either a) an ‘internal’ issue that must be addressed at an individual level, or b) part of a larger issue that revolves around a societal adaptation appear to be two sides of the same coin.

To begin, this study has shown that short-sighted reliance on individual or societal theories have resulted in incomplete understanding, program design and assessments of resilience. These may be divided into individual and environmental factors. The individual factors include developing competencies, experiences and maturity. Environmental factors include program nature, culture, structure, workload and the availability, nature and quality of support. Based on the data, there is a lack of longitudinal formal programs scaffolded on a spiral curriculum along the length of the course—compromising opportunities for learners to apply their new skills and competencies. This point has not been lost on more recent studies that have sought to combine different theories to better reflect a more holistic perspective of resilience. In this study, it is proposed in consultation with the RToP lens that programs should also account for elements in the Innate and Relational Rings. Moral distress, conflicts with spiritual beliefs, as well as a lack of a strong support network of family and friends, threaten resilience and predispose to *the costs of caring*—a concept extending beyond mere compassion fatigue that, when left unattended, can lead to burnout.^20,22-24^

Indeed, resilience is an evolving notion that is buffeted by a myriad of personal, academic, psycho-emotional, research and clinical considerations along the residency program. These *costs of caring* underline the need for personalized, accessible, longitudinal and timely support provided by trained mentors in a psychologically safe environment. It is therefore proposed that resilience may be better introduced and built through a mentoring approach. Current approaches revolving around workshops on mindfulness practices, self-compassion, reframing, self-awareness, self-care, stress management skills and maximizing work-life balance and well-being that also encourage realistic goal setting, managing expectations and nurturing reflection and gratitude tend to be generalized and not focused on the needs of the particular resident.^4,15,20,21,23,27,30,34,37,40,80-85^ Structured, organization-led, once-off or one-size-fits-all resilience training do not cater for the resident’s immediate needs nor provide timely interventions.^4,18,21,27,43,84,86-89^ Even social support networks are rarely supported by trained facilitators who can provide appropriate and meaningful support.^24,84,88^ Other organizational initiatives, such as flexible scheduling and improved working hours, are also generalized and not focused on individual needs.^15,21,43^

Mentoring offers residents a means of timely, personalized and longitudinal support from trained faculty as residents confront the *costs of caring*. Current mentoring practices suggest that such support can be culturally sensitive, resource-appropriate, sustainable and clinically relevant, particularly when integrated with e-portfolio use.^91-94^ It is through mentoring and its personalized mix of role modelling, advising, teaching, coaching, supervision, networking and remediation that shape how the resident sees, feels and acts like a professional, or their professional identity formation.^58,95,96^ Nurturing a professional identity where residents embrace and commit to a role and set of responsibilities will guide meaning-making, renewal of goals and encourage renewed vigor and support for resilience. Professional identity formation will also guide a resident’s conduct with patients, families and other professionals and further build much needed experiences and insights that will only strengthen their resolve when effectively guided by an invested and trained mentor.^61,97-99^

### Limitations

This review is constrained by the exclusion of non-English papers, studies focusing on other health care professionals (eg, nursing, allied health, pharmacy, dentistry, etc.) and research published prior to the year 2000. The predominance of articles sourced from North America emerging from a specific cultural context raises questions about the generalizability of these findings to other practice settings, considering the contextual factors that influence one’s personhood, practice setting and the wider *costs of caring*. Further studies in resilience training in residency are thus proposed, focusing on different countries and cultures to better delineate the cultural influences affecting resilience, burnout and the *costs of caring*. Additionally, the reliance on self-reported scales and tools grounded in *“Cartesian reductionism and Newtonian principles of linearity”*, which do not account for the dynamic and evolving nature of resilience and burnout, highlights the need for further systematic reviews and prospective research studies.^100,101^

## Conclusion

Taking the notion of a structured mentored approach to supporting resilience and drawing individual and organizational efforts further as a means to improve professional identity formation and ward against the *costs of caring* will require careful study. Fortunately, tools are readily available to facilitate this process. Evaluating their viability and developing a framework to build resilience will be the focus of coming work.

## Supplemental Material

Supplemental Material - A Framework to Combat Burnout Amongst Residents Through Fostering Resilience: A Systematic Review Supplemental Material for A Framework to Combat Burnout Amongst Residents Through Fostering Resilience: A Systematic Review by Le Xuan Shannon Tan, Jiayan Ding, Timothy Xuxian Neo, Vincent Sixian Chen, Charmaine Shi Mei Lee, Nur Amira Binte Abdul Hamid, Yun Ting Ong and Lalit Kumar Radha Krishna in American Journal of Hospice and Palliative Medicine®

Supplemental Material - A Framework to Combat Burnout Amongst Residents Through Fostering Resilience: A Systematic Review Supplemental Material for A Framework to Combat Burnout Amongst Residents Through Fostering Resilience: A Systematic Review by Le Xuan Shannon Tan, Jiayan Ding, Timothy Xuxian Neo, Vincent Sixian Chen, Charmaine Shi Mei Lee, Nur Amira Binte Abdul Hamid, Yun Ting Ong and Lalit Kumar Radha Krishna in American Journal of Hospice and Palliative Medicine®

## Data Availability

All data generated or analyzed during this study are included in this published article and its supplementary files*.

## Appendix

### Abbreviations

PCC: Population, Context, Concept
RToP: Ring Theory of Personhood
SEBA: Systematic Evidence-Based Approach
