# Supplementary material for: Combating Burnout Amongst Residents Through Fostering Resilience: A Systematic Review
Source: Am J Hosp Palliat Care. 2025 Mar 28;43(4):438–48. doi: 10.1177/10499091251331150 (PMC12926512; doi:10.1177/10499091251331150)
Supplement: Supplemental Material - A Framework to Combat Burnout Amongst Residents Through Fostering Resilience: A Systematic Review [file sj-pdf-2-ajh-10.1177_10499091251331150.pdf]

Additional File 2. Examples of Search Strategy Applied to Database Search

PubMed	
After publication date filter (from 1st Jan 2000 to 4th Nov 2024) and language filter (English)<br><br>(“Resilience, Psychological"[Mesh] OR “resilien*”[tiab] OR grit[tiab] OR tenaci*[tiab] OR persisten*[tiab] OR perserve*[tiab]) AND (((“medic*”[tiab] OR “clinic*”[tiab] OR “Medicine”[Mesh]) AND (“student*”[tiab] OR “school*”[tiab])) OR “Students, Medical”[Mesh] OR “Schools, Medical”[Mesh] OR “doctor*”[tiab] OR “physician*”[tiab] OR “Physicians”[Mesh] OR “resident”[tiab] OR “residency”[tiab]) NOT (hous*[tiab] OR patient*[tiab] OR bacteri*[tiab] OR persistent[tiab])	4163
**Embase**	
After publication date filter (from 1st Jan 2000 to 4th Nov 2024) and language filter (English)<br><br>('psychological resilience'/mj OR “resilien*”:ti,ab OR grit:ti,ab OR tenaci*:ti,ab OR persisten*:ti,ab OR perserve*:ti,ab) AND (((“medic*”:ti,ab OR “clinic*”:ti,ab OR “Medicine”/mj) AND (“student*”:ti,ab OR “school*”:ti,ab)) OR 'medical student'/mj OR 'medical school'/mj OR “doctor*”:ti,ab OR “physician*”:ti,ab OR 'physician'/mj OR “resident”:ti,ab OR “residency”:ti,ab) NOT (hous*:ti,ab OR patient*:ti,ab OR bacteri*:ti,ab OR persistent:ti,ab) NOT (hous*:ti,ab OR patient*:ti,ab OR bacteri*:ti,ab OR persistent:ti,ab)	4801
**Scopus**	

After publication date filter (from 1st Jan 2000 to 4th Nov 2024) and language filter (English)<br><br>(“resilien*” OR grit OR tenaci* OR persisten* OR perserve*) W/3 (((“medic*” OR “clinic*” OR “Medicine”) W/3 (“student*” OR “school*”)) OR “doctor*” OR “physician*” OR “resident” OR “residency”) AND NOT (hous* OR patient* OR bacteri* OR persistent)	787

Additional File 3. Summaries of Included Articles Generated by ChatGPT 4o

No.	Author	Year	Title	Study Aim	Methods	Key Findings	Conclusion
1	O'Connell et al.	2020	A Workshop to Reflect on Personal Resilience in Emergency Medicine Residents: Applying the Connor-Davidson	The study highlights growing concerns about burnout among emergency medicine (EM) residents, a condition characterized by	The workshop included 90-minute sessions during weekly conferences, starting with self-assessments via the Connor-Davidson	Residents scored below the US general population in resilience, with the lowest scores observed in postgraduate year 2 (PGY-2) residents.	The study emphasized the need for more correlation data between resilience scores and burnout and suggested that workshops

			Resilience Scale, Visual Explorer, and the Critical Incident Questionnaire to Support Introspection	emotional exhaustion, depersonalization, and reduced effectiveness. Burnout's impact on mental health and patient care has led to calls for incorporating wellness and resilience training into medical education. This study introduced a workshop to address resilience using structured	Resilience Scale (CD-RISC). Residents reflected on resilience through imagery using the Visual Explorer toolkit and discussed their personal insights in small groups. Facilitators used structured reflective questions to deepen engagement.	The Critical Incident Questionnaire revealed that residents valued peer discussions and visual tools, though they felt less engaged during the didactic segments.	fostered valuable self-reflection and peer support.

				introspection and peer engagement methods.			
2	Chew QH et al.	2019	A Cross-Sectional Study of Burnout and its Associations with Learning Environment and Learner Factors among Psychiatry Residents within a National Psychiatry Residency Programme	Psychiatry residents experience high burnout rates, yet few studies address the interplay between burnout and learning contexts, resilience, and coping mechanisms. This research aimed to fill that gap by exploring these factors in a national residency	A cross-sectional study surveyed 93 psychiatry residents using the Oldenburg Burnout Inventory, Perceived Stress Scale, and other validated tools to assess resilience, stigma, and coping. Comparisons were made between	Over half of the residents met the burnout criteria, with correlations identified between burnout and greater perceived stress, poorer perceptions of the learning environment, and avoidance-focused coping strategies. Non-burnout	Burnout was strongly linked to stress, stigma, and environmental factors. The findings highlight the need for tailored interventions that address both learner-specific and systemic stressors in residency

				program.	burnout and non-burnout groups.	residents used more problem-focused coping methods.	programs.
3	Bird, Martinchek, Pincavage	2017	A Curriculum to Enhance Resilience in Internal Medicine Interns	Burnout poses significant challenges in medical education, particularly for internal medicine (IM) interns who face intense stress during their early training years. This study developed and implemented a curriculum	The curriculum comprised four workshops delivered by chief residents, focusing on setting realistic goals, managing expectations, processing clinical stress, and fostering gratitude. Sessions incorporated	While participants valued the sessions and reported increased comfort discussing stress, burnout, and medical errors, resilience scores showed a slight decline post-intervention. Qualitative feedback indicated a	The program demonstrated feasibility and was well-received, but its limited impact on resilience scores suggests the need for further refinement and evaluation of such curricula.

				designed to teach resilience skills.	lectures, discussions, reflection, and skill-building exercises. Surveys assessed participants' resilience before and after the program.	sense of community and peer support fostered during sessions.	
4	Bird, Pincavage	2016	A Curriculum to Foster Resident Resilience	Medical trainees face significant stress, often leading to burnout and its associated mental health challenges. Enhancing resilience is considered a	A three-session curriculum addressed critical resilience-building strategies, including goal-setting, stress processing, and gratitude	Participants noted increased awareness of stress management techniques and appreciated the opportunity to share challenges with peers in	This curriculum underscored the importance of incorporating resilience training into medical education to support trainees’ mental

				promising strategy to mitigate these effects.	exercises. Sessions were structured around small group discussions, reflective writing, and practical exercises, emphasizing peer support.	a structured environment. Facilitators reported high engagement during discussions.	health and professional growth.
5	Harsh et al.	2019	A New Perspective on Burnout: Snapshots of the Medical Resident Experience	Burnout in residency training affects professional performance and personal well-being, yet qualitative studies exploring the	Eight residents from internal and family medicine programs participated, capturing photographs representing their burnout experiences. Semi-	Residents described burnout as pervasive, inducing emotional exhaustion, self-doubt, and disengagement. Coping strategies included	The study revealed the multidimensional impact of burnout on residents' lives and emphasized the need for intervention

				resident experience are limited. This study used a novel approach involving photography to gain deeper insights into residents' perceptions of burnout.	structured interviews followed, exploring themes like stress, self-doubt, and coping strategies. Data were analyzed using the Transcendental Phenomenological Method.	seeking support from colleagues, engaging in self-care, and reframing challenges as growth opportunities.	s fostering resilience and peer support.
6	Kiesewetter, Huber	2021	A Primer of an In-Depth Resilience Status for German Medical Graduates	Resilience is crucial for medical graduates transitioning into practice. However, its components and correlations	A cross-sectional survey of 610 Bavarian medical graduates used the German version of	Graduates showed high overall resilience but struggled with handling negative emotions.	The findings point to the need for resilience-focused training programs that address

				with stress and job satisfaction require further exploration.	the Connor-Davidson Resilience Scale and other tools to assess resilience, stress, job satisfaction, and scientific competence.	Resilience correlated positively with job satisfaction and negatively with perceived stress, highlighting gaps in emotional coping skills.	specific emotional challenges encountered in early medical careers.
7	Zhou, Money, Bower	2019	A Qualitative Study Exploring the Determinants, Coping, and Effects of Stress in United Kingdom	UK trainee doctors report high stress levels due to workload, systemic pressures, and personal expectations. This study explored the	Focus groups with 44 trainees from various specialties identified key stressors and coping strategies through thematic analysis.	Determinants included workload, feelings of undervaluation, and uncertainty in career progression. Coping strategies ranged from reflection	The study highlighted the need for organizational and individual-level interventions to mitigate stress and promote

			Trainee Doctors	determinants, coping mechanisms, and effects of stress in this group.		and mindfulness to seeking a better work-life balance.	resilience among trainees.
8	Benzie et al.	2023	A Self-Selecting Prophecy: Prevalence of Burnout in Surgical Fellows	Despite high burnout rates among surgical residents, little is known about burnout prevalence in surgical fellows. This study aimed to fill this gap.	Surveys were distributed to 196 surgical fellows, assessing burnout, stress, grit, life satisfaction, and self-efficacy using validated tools.	Burnout prevalence was 8.4%, significantly lower than among surgical residents. Factors like mentorship, specialization, and autonomy were protective, while stress and lower self-esteem correlated with burnout.	The low burnout rates suggest a self-selection effect in fellowship applicants and emphasize the role of structured support during advanced training.
9	Seo et	20	Addressin	Burnout	Twenty-one	Most studies	Resilience

	al.	21	g the Physician Burnout Epidemic with Resilience Curricula in Medical Education: A Systematic Review	among medical trainees has led to increased interest in resilience-based interventions. This systematic review synthesized evidence on resilience curricula in medical education.	studies involving resilience training at undergraduate and postgraduate levels were reviewed. The interventions varied widely in design and outcomes.	reported modest improvements in resilience, though some noted negative effects. Interventions lacked consistency, limiting generalizability.	training holds promise but requires standardization and rigorous evaluation to optimize its impact on trainees.
10	Tarana Lucky et al.	2022	Are We Gritty Enough? The Importance of Grit in O&G Training	This study investigates the role of grit—a combination of passion and perseverance—as a	A cross-sectional survey involving 751 participants, categorized as core trainees,	Fellows exhibited higher grit and lower burnout levels compared to trainees. Grit scores	Grit serves as a protective factor against burnout. The study recommends targeted

				protective factor against burnout in obstetrics and gynecology (O&G) trainees. Grit is theorized to not only enhance resilience but also contribute to career progression and thriving, areas that are underexplored in the context of O&G training, where stress levels are	advanced trainees, and Fellows, was conducted using the Short Grit Scale and the Oldenburg Burnout Inventory. Data were analyzed through Pearson's correlation and logistic regression to examine the relationships between grit, burnout, and seniority levels.	negatively correlated with burnout, with a moderate negative correlation observed. Seniority and grit levels were the strongest predictors of burnout reduction.	interventions for high-risk groups, especially early-career trainees, to foster grit and reduce burnout.

				high.			
11	Martinchek, Bird, Pincavage	2017	Building Team Resilience and Debriefing After Difficult Clinical Events	Leadership and resilience are critical skills for medical team leaders who frequently encounter stressful events. This curriculum aims to equip healthcare team leaders, particularly senior residents, with the skills to lead debriefings and build team	The curriculum was structured into two interactive workshops, focusing on leadership strategies and team-based debriefing exercises. Participants included senior residents and fellows who completed pre- and post-workshop surveys.	Participants reported increased confidence in managing team discussions after difficult clinical events. They found the debriefing tools practical and appreciated the opportunity to reflect on their leadership skills.	The workshops were effective in teaching resilience and leadership strategies. Institutions should integrate such curricula to address both individual and team well-being in healthcare settings.

				resilience after challenging clinical situations.			
12	McKinley, Boland, Mahan	2017	Burnout and Interventions in Pediatric Residency: A Literature Review	Pediatric residents experience high rates of burnout, attributed to the emotionally demanding nature of their work. This review synthesizes available literature on burnout prevalence, risk factors, and intervention strategies in pediatric	A systematic review of studies published between 2005 and 2016 was conducted using multiple databases. Inclusion criteria focused on pediatric residency, burnout, and intervention strategies.	Burnout rates among pediatric residents ranged from 17% to 67.8%, with emotional exhaustion and depersonalization being the most common dimensions. Interventions such as mindfulness training, work-hour limitations, and	Effective burnout interventions require a multifaceted approach, incorporating both systemic changes and individual coping strategies. Program directors should prioritize evidence-based initiatives

				residency programs.		psychosocial support showed mixed effectivenes s.	tailored to pediatric trainees.
1 3	Hyojung Shin	20 23	Efficacy of the Online Mindful Self-Compassi on Program for Surgical Trainees	Burnout among surgical trainees is prevalent, with the COVID-19 pandemic exacerbating stress levels. Self-compassion has emerged as a key resilience factor, but its application in surgical training remains	A single-arm pilot study with 15 surgical trainees assessed the impact of a six-week online Mindful Self-Compassion (MSC) program. Burnout, anxiety, stress, and resilience were measured pre- and post-	Participants reported significant reductions in burnout, anxiety, and stress, alongside improvemen ts in resilience and life satisfaction. Gains in self-compassion were linked to reduced burnout and higher resilience	The MSC program is a feasible and effective tool to mitigate burnout and enhance resilience in surgical trainees. Institutions should consider incorporatin g self-compassio n training into their

				underexplored.	intervention using validated scales.	scores.	wellness curricula.
14	Elyse Guran et al.	2022	Evaluation of Psychological Impact of COVID-19 on Anesthesiology Residents in the United States	The COVID-19 pandemic introduced unprecedented stressors for anesthesiology residents, including increased work hours and exposure to high-risk environments. This study explored the pandemic's psychological impact on this vulnerable group.	A survey of 111 anesthesiology residents across 159 US residency programs used tools such as DASS-21, aMBI, and BRCS to assess depression, anxiety, stress, and burnout levels.	Burnout prevalence was 71%, with 80% experiencing emotional exhaustion. Prior mental health diagnoses and increased work hours were significant predictors of higher stress and burnout.	COVID-19 heightened psychological distress among anesthesiology residents, emphasizing the need for targeted mental health interventions and systemic support.

15	Wood, Egan, Ange et al.	2020	Association of Self-Reported Burnout and Protective Factors in Single Institution Resident Physicians	This study investigates the role of grit, resilience, social support, and psychological flexibility as protective factors against burnout in medical residents. The goal was to determine which factors most effectively mitigate burnout and how they are utilized during residency.	A mixed-methods approach was used, including an online survey of 268 residents from 20 residency programs and interviews with 13 residents. Burnout was assessed using the Maslach Burnout Inventory (short form), while protective factors were measured	Grit was the strongest protective factor, showing a significant negative correlation with burnout. Social support from peers was highly valued, with many residents indicating it as their primary coping mechanism. Psychological flexibility emerged as another critical factor,	While resilience programs are widely promoted, grit and social support are equally important in combating burnout. Residency programs should foster peer networks and teach psychological flexibility to improve well-being.

					using validated scales for grit, resilience, and psychological flexibility.	enabling residents to adapt and manage stress effectively.	
16	Hategan, Riddell	2020	Bridging the Gap: Responding to Resident Burnout and Restoring Well-being	Resident burnout is a growing concern, with systemic and personal factors contributing to high stress levels. This study evaluates a pilot program aimed at reducing	The program was conducted at McMaster University and included voluntary participation from psychiatry residents. Peer groups provided confidential spaces for reflection, while the online curriculum	Of the participants, 48% reported burnout at enrollment. After engaging with the program, a 50% reduction in perceived stress levels was reported. Peer groups were rated as the most	Structured peer support combined with digital resources effectively reduces burnout in residency training. Institutions should integrate such multi-faceted approaches into their wellness

				burnout through a triad of interventions: peer groups, an electronic resilience curriculum, and wellness newsletters.	and newsletters offered resilience strategies. Feedback was collected through surveys and qualitative evaluations.	beneficial component, with senior residents showing the highest levels of engagement.	strategies.
17	Herodotos Ellinas, Elizabeth Ellinas	2020	Burnout and Protective Factors: Are They the Same Amid a Pandemic?	The COVID-19 pandemic introduced unique stressors, potentially altering the effectiveness of known protective factors against burnout. This commentary	The study builds on prior research by Wood et al., exploring protective factors in residents during normal circumstances and speculating on their	Grit and social support remained significant protective factors, but their effectiveness varied. Psychological flexibility gained prominence as residents	Pandemic stress requires recalibration of wellness strategies. Enhancing psychological flexibility and fostering peer support may better

				examines how grit, social support, psychological flexibility, and resilience operate during crises.	applicability during the pandemic. Literature on burnout and resilience in crises was reviewed.	needed to adapt to rapidly changing conditions. Resilience, while essential, showed diminished impact in the face of overwhelming systemic stressors.	address burnout in extreme conditions like COVID-19.
18	Franco et al.	2022	Burnout and Resilience of Internal Medicine Physician Trainees in a Tertiary Government Hospital in the	Internal medicine trainees were on the frontlines during the pandemic, facing significant challenges that heightened	A mixed-methods approach included a survey of 146 trainees using the Maslach Burnout Inventory and Connor-Davidson	Burnout prevalence was 4%, with 40% of trainees classified as engaged. Resilience significantly reduced burnout risk, but its	While resilience mitigates burnout, systemic changes addressing workload and emotional support are critical.

			Philippines During the COVID-19 Pandemic	their risk of burnout. This study examines burnout prevalence, resilience levels, and coping strategies among these trainees.	Resilience Scale. Focus group discussions and interviews explored sources of stress and coping mechanisms.	protective effect diminished with increased exposure to patient deaths. Common stressors included changes in workflows, telemedicine fatigue, and the emotional toll of patient loss. Coping mechanisms included self-care, social connections, and preserving personal	Institutions should prioritize trainee well-being through tailored interventions.

						boundaries.	
19	Winkel et al.	2024	Burnout and Well-Being in Trainees: Findings From a National Survey of US Obstetrics and Gynecology Residents	Obstetrics and gynecology (OB/GYN) residents face high rates of burnout, depression, and anxiety due to strenuous working conditions. This study evaluates these issues on a national scale and identifies personal and systemic factors contributing to well-	A survey of 3,741 residents was conducted following the 2022 in-training examination. Burnout, resilience, depression, and anxiety were measured using the Maslach Burnout Inventory and Connor-Davidson Resilience Scale, among other tools.	Burnout prevalence was 64.8%, with depression at 57.2% and anxiety at 70.9%. Women and nonbinary residents reported higher levels of mental health concerns compared to men. Programs that did not prioritize well-being had significantly worse outcomes,	Comprehensive well-being programs are critical to addressing burnout in OB/GYN residency. Targeted interventions should consider gender and program culture to improve resident mental health and professional satisfaction.

				being.		with 87.8% of residents reporting burnout.	
20	Yun Song et al.	2020	Can We Coach Resilience? An Evaluation of Professional Resilience Coaching as a Well-Being Initiative for Surgical Interns	Burnout among surgical interns is a pressing issue, with nearly 60% at risk upon entering their residency programs. The study evaluates the effectiveness of a year-long resilience coaching program aimed at fostering	A mixed-methods approach included pre- and post-coaching surveys using the Brief Resilience Scale and the Abbreviated Maslach Burnout Inventory. Additionally, 16 participants engaged in semi-structured interviews to	Participants showed significant improvement in resilience scores post-coaching (3.8 to 4.2, p = 0.002). However, burnout indicators remained unchanged, and participants expressed concerns about the durability of a one-year intervention.	While the program positively influenced resilience, a longitudinal approach may be necessary to sustain its benefits and effectively tackle burnout throughout surgical training.

				well-being and reducing burnout during the challenging transition into residency.	explore their experiences.	The program provided a “safe space” for introspection and was appreciated for its flexibility in addressing individual needs.	
21	Johnson et al.	2020	Can We Prepare Healthcare Professionals and Students for Involvement in Stressful Healthcare Events? A Mixed-Methods	Stressful healthcare events, such as adverse outcomes and high-stakes decision-making, significantly impact healthcare professionals' mental health. This	The study employed a before-and-after design with 66 participants from various disciplines. The intervention included a 3.5-hour workshop and one-on-one	Significant improvements were observed in confidence ($d = 1.55, p < 0.001$), knowledge ($d = 0.86, p < 0.001$), and resilience ($d = 0.65, p < 0.001$) across all	The intervention effectively enhanced resilience and preparedness for adverse events. However, further research is needed to confirm its

			Evaluation of a Resilience Training Intervention	study evaluated a resilience training intervention tailored to these challenges.	coaching, assessing confidence, knowledge, and resilience at four time points using quantitative and qualitative methods.	time points. Participants valued peer engagement and personalized coaching but highlighted the tension between mandatory and voluntary training delivery.	efficacy in different healthcare contexts.
22	Budisavljevic et al.	2023	Correlation Between Psychological Resilience and Burnout Syndrome in Oncologists Amid the COVID-19	Oncologists face high burnout risks due to their demanding roles, with the COVID-19 pandemic exacerbating stress. This study investigated	A cross-sectional survey using the Oldenburg Burnout Inventory and Brief Resilience Scale was distributed to 130	High burnout was reported in 86% of respondents, while 77% displayed moderate to high resilience. Resilience negatively	Psychological resilience acts as a protective factor against burnout. The study advocates for resilience-

			Pandemic	the relationship between psychological resilience and burnout among Croatian oncologists during the pandemic.	oncologists, achieving a 57.7% response rate. Data analyzed burnout levels and resilience correlations.	correlated with burnout ($r = -0.54$, $p < 0.001$), particularly with the exhaustion dimension.	building interventions to support oncologists during crises like pandemics.
23	Brennan and McGrady	2015	Designing and Implementing a Resiliency Program for Family Medicine Residents	Family medicine residents often experience burnout due to long hours and high demands. This program aimed to build resilience through self-	The program included interactive sessions on self-care, time management, and mindfulness. Structural changes, such as improved nutrition options and exercise	The program was well-received, with increased consumption of nutritious foods and improved exercise habits. Participants reported decreased stress reactions	Comprehensive resilience programs can effectively foster well-being and prevent burnout in family medicine residency programs.

				awareness, coping skills, and systemic wellness improvements.	equipment, were also introduced. Resident feedback and behavioral changes were assessed qualitatively.	and enhanced work-life balance.	
24	Mullins et al.	2020	Do Internal or External Characteristics More Reliably Predict Burnout in Resident Physicians? A Multi-Institutional Study	This study explores whether burnout in surgical residents is more influenced by internal characteristics (resilience, mindfulness, emotional intelligence) or external	Surveys were distributed to 164 general surgery residents across seven institutions. Variables included the Maslach Burnout Inventory, Connor-Davidson	While internal characteristics correlated with lower burnout on bivariate analysis, multivariable models revealed that work engagement and job resources were the	External factors, particularly workplace resources and engagement, play a more significant role in mitigating burnout than internal traits.

				factors (work environment, resources).	Resilience Scale, and Utrecht Work Engagement Scale.	most significant protective factors against burnout.	Programs should prioritize improving work environments and resources.
25	Tan et al.	2023	Drivers of Well-Being and Burnout in Anesthesiology Residents	Anesthesiology residents face high rates of burnout, with nearly 60% reporting symptoms. This study explores factors driving well-being and burnout among anesthesiology residents using the Areas of	Semi-structured interviews were conducted with CA-2 and CA-3 residents from UCSF. The AW model guided the analysis, examining themes such as workload, control, community, and reward.	Key challenges included work overload, lack of control over schedules, insufficient rewards, and a sense of isolation exacerbated by the COVID-19 pandemic. Residents highlighted the	Systemic interventions are critical to improving anesthesiology residents' well-being. The study emphasizes the need for tailored strategies addressing workload, community building, and

				Worklife (AW) model, focusing on systemic and individual influences.	Data were analyzed through thematic analysis for emerging patterns.	importance of peer support, professional identity formation, and mentorship in mitigating burnout. Positive relationships with faculty and autonomy were also identified as protective factors.	professional development.
26	Bursch et al.	2019	Evaluation of Curriculum to Teach Resilience Skills to Neurology	Neurology residents experience high stress and burnout rates. This study evaluates a	Twenty-two residents participated in five 1-hour sessions covering emotion regulation,	Half of the residents showed moderate-to-high emotional exhaustion at baseline.	The resilience training curriculum was well-received and showed

			Residents	resilience training curriculum adapted from military family resilience programs, focusing on skills like boundary management and reflective practices.	reflective narrative, communication with distressed individuals, and coping with trauma. Surveys measured burnout, resilience, and self-efficacy pre- and post-intervention.	Post-training, significant improvements were noted in self-efficacy for stress management and identifying patient-related grief and trauma. Resident satisfaction with the curriculum was high (71%), and qualitative feedback highlighted its relevance and practicality.	positive impacts on self-efficacy. Future research should explore long-term effects on burnout and resilience.
2	Brennan	20	Effects of	Burnout	The study	Immediately	Short-term

7	et al.	19	a Resiliency Program on Burnout and Resiliency in Family Medicine Residents	remains a significant issue for family medicine residents, with limited evidence-based programs addressing this challenge. This study tested an eight-hour resilience program aimed at reducing burnout and enhancing resilience.	involved 32 residents divided into intervention (19) and control (13) groups. The intervention group attended sessions focused on mindfulness, time management, coping skills, and maintaining work-life balance. Burnout and resilience were measured using the Maslach Burnout	post-intervention, participants showed significant reductions in emotional exhaustion and depersonalization compared to the control group. Long-term follow-ups revealed sustained improvements in resilience but no significant changes in burnout markers beyond the initial	resilience programs can effectively reduce burnout and improve coping skills, but additional measures are needed for long-term sustainability.

					Inventory and Connor-Davidson Resilience Scale pre- and post-intervention, with follow-ups at one and two years.	results.	
28	Winkel et al.	2024	Evaluating the Impact of Coaching Through the Transition to Residency	Transitioning to residency is often marked by stress, burnout, and professional identity challenges. This study evaluates the impact of coaching programs on resilience, professional	A cohort study compared two groups: coached residents and uncoached controls. Coaches, trained faculty members, conducted semi-structured	Coached residents reported higher resilience, professional fulfillment, and career satisfaction compared to their uncoached counterparts. Burnout rates were lower among	Coaching is a valuable tool for supporting residents during the transition to residency, particularly for those experiencing burnout. Institutions should integrate coaching

				development, and well-being during the transition to residency.	meetings with residents during their first year. Surveys assessed professional fulfillment, resilience, and burnout.	coached residents (51% vs. 69%). The program’s impact was more pronounced among residents experiencing higher burnout levels.	into graduate medical education.
29	Loewenthal et al.	2021	Evaluation of a Yoga-Based Mind-Body Intervention for Resident Physicians: A Randomized Clinical Trial	Mind-body interventions (MBIs), such as yoga, are effective in mitigating burnout. This study evaluates the feasibility and efficacy of a yoga-	A randomized controlled trial with 56 residents (38 intervention, 18 control) assessed the impact of six weekly yoga sessions on mindfulness,	Participants in the RISE program reported significant improvements in mindfulness, stress, burnout, and resilience. However, logistical	The yoga-based intervention was effective in improving psychological health, but virtual delivery may enhance feasibility

				based MBI (RISE program) tailored for medical residents.	stress, burnout, and resilience. Measures included self-reported surveys at baseline, post-program, and two-month follow-up.	challenges, such as attendance barriers due to work schedules, limited the feasibility of in-person delivery.	and engagement for busy residents.
30	Guran et al.	2022	Evaluation of Psychological Impact of COVID-19 on Anesthesiology Residents in the United States	The COVID-19 pandemic brought unprecedented stress to anesthesiology residents, with factors such as exposure risk, workload surges, and reduced	A survey incorporating DASS-21, Abbreviated Maslach Burnout Inventory (aMBI), and Brief Resilient Coping Scale (BRCS) was distributed to	High levels of burnout (71%) were observed, with emotional exhaustion affecting 80%. Depression and anxiety prevalence were 42% and 24%,	COVID-19 significantly worsened burnout and mental health issues among anesthesiology residents. Resilience and institutional

				training opportunities exacerbating psychologic al distress.	159 residency programs. 111 residents completed the survey.	respectively. Residents with prior mental health diagnoses were more susceptible to stress and burnout.	support emerged as critical factors for mitigating these effects.
3 1	Lebares et al.	20 21	Exploratio n of Individual and System-Level Well-being Initiatives at an Academic Surgical Residency Program: A Mixed-Methods Study	Physician well-being is critical, yet data on effective well-being programs for surgical trainees are limited. This study explores individual and workplace factors influencing	A mixed-methods approach included a survey of 98 surgical trainees and a focus group with 9 residents. The study assessed psychosocial risk and resilience using validated	Women were more likely to report high depersonaliz ation and lower mindfulness than men. Barriers included time constraints and organization al inefficiencies	Multilevel well-being programs tailored to individual and organizatio nal needs are essential. Addressing specific barriers can enhance their effectivene

				well-being and differences by gender.	scales and examined perceptions of institutional well-being initiatives.	. Key beneficial factors included effective scheduling, mindfulness training, and social support.	ss.
32	Nituica et al.	2021	Factors Influencing Resilience and Burnout Among Resident Physicians - A National Survey	Residency training creates high burnout risk, negatively impacting residents' health and patient care. This study examined resilience and its relationship to burnout across U.S. resident	A cross-sectional survey assessed 682 residents using the Connor-Davidson Resilience Scale (CD-RISC) and Maslach Burnout Inventory. Demographics and	Resilience positively correlated with personal achievement and negatively with emotional exhaustion and depersonalization. Males and senior residents exhibited	Enhancing resilience through structured training and support systems can mitigate burnout and improve resident well-being.

				physicians.	institutional support factors were also analyzed.	higher resilience. Institutional and family support were significant protective factors.	
33	Martinelli et al.	2019	Family Comes First: A Pilot Study of the Incorporation of Social Support Into Resident Well-being	Social support is vital for resident well-being, but many residents struggle to communicate their professional stress to family and friends. This study evaluates a program educating	The Family Anesthesia Experience (FAX) program included interactive sessions and simulations for SPs. Surveys assessed SPs' understanding and residents' perceptions	SPs' understanding of residents' roles significantly improved. Residents reported enhanced communication with SPs and reduced stress, though burnout levels increased	Engaging SPs through educational programs can enhance social support and reduce stress, though sustained interventions may be necessary to address burnout

				residents' support persons (SPs) about their roles.	of support pre- and post-program.	toward the end of the study.	effectively.
34	Buck et al.	2019	Family Physician Burnout and Resilience: A Cross-Sectional Analysis	Family physicians face high burnout rates due to workload and systemic pressures. This study explored the relationship between resilience and burnout, with a focus on psychological flexibility and workplace stress.	A survey of 295 family medicine physicians assessed burnout, resilience, and workplace stress using validated scales. Regression analysis examined predictors of well-being.	Resilience negatively correlated with burnout and positively with psychological flexibility. Higher resilience was associated with supportive work environments and healthy habits.	Targeted interventions enhancing resilience and reducing workplace stress are crucial for mitigating burnout in family medicine.

35	Lebares et al.	2021	Flourishing as a Measure of Global Well-being in First-Year Residents: A Pilot Longitudinal Cohort Study	Physician well-being is critical, yet validated tools for assessing it are lacking. This study evaluates the concept of "flourishing" as a measure of global well-being in first-year residents, exploring its relationship with resilience and burnout.	A cohort of 45 first-year residents participated in Enhanced Stress Resilience Training (ESRT). Flourishing was measured using the Mental Health Continuum (MHC), with assessments at baseline, post-ESRT, and year-end. Resilience and burnout metrics included	Flourishing was positively correlated with mindfulness and workplace support, while negatively correlated with emotional exhaustion, stress, and depressive symptoms. Participants' flourishing peaked post-ESRT but declined slightly by year-end.	Flourishing is a valid, multi-dimensional measure of well-being. Using it to evaluate interventions like ESRT can help track and improve resident well-being longitudinally

					mindfulness, emotional exhaustion, and stress.		
36	Tan et al.	2021	Fostering Resilience in Junior Doctors: Learning from Resilience Traits and Coping Strategies of Senior Physicians	Junior doctors face high burnout rates compared to senior physicians. This study explores resilience traits and coping strategies among senior doctors to identify lessons for fostering resilience in junior doctors.	A mixed-methods study surveyed 31 senior pediatricians using the Connor-Davidson Resilience Scale (CD-RISC) and Maslach Burnout Inventory. Semi-structured interviews with 15 participants explored resilience traits and	Senior physicians demonstrated higher resilience and lower burnout rates. Key traits included self-awareness, perseverance, and optimism. Coping strategies like prioritization, reflective practice, and seeking peer support	Resilience in senior physicians stems from specific traits and strategies, which junior doctors can adopt through mentorship and structured training programs.

					strategies.	were common.	
37	Rodriguez-Unda et al.	2023	Global Resilience in Plastic Surgery Study (GRIPS): Resilience is Associated with Lower Burnout Rates	Burnout is prevalent among plastic surgery trainees, yet the protective role of resilience remains underexplored. This international study assesses resilience and burnout among trainees across 24 countries.	A survey of 175 trainees included the Connor-Davidson Resilience Scale (CD-RISC) and Maslach Burnout Inventory. Multivariate regression examined factors affecting burnout and resilience.	Burnout was reported by 44.5% of respondents. Trainees with higher resilience scores and access to wellness programs showed significantly lower burnout rates. Increased work hours correlated with higher burnout.	Resilience mitigates burnout in plastic surgery trainees. Programs should integrate resilience-building initiatives and reduce workload to enhance well-being.
38	Ogunyemi et al.	2022	Graduate Medical Education-	Residency programs are	Over three years, 271 residents	Emotional exhaustion and	Longitudinal assessmen

			Led Continuous Assessment of Burnout and Learning Environments to Improve Residents' Well-being	addressing burnout and well-being, yet longitudinal approaches using systematic interventions are rare. This study evaluates the impact of continuous assessment and targeted interventions on resident well-being.	from six programs completed five assessments using the Maslach Burnout Inventory and a clinical learning environment survey. Interventions were designed and implemented based on findings using Plan-Do-Study-Act (PDSA) cycles.	depersonalization decreased over time, while personal accomplishment improved. Positive perceptions of clinical supervision were associated with better outcomes. Internal Medicine residents showed the most significant improvements.	ts combined with targeted interventions effectively improve resident well-being. Scaling such initiatives across specialties could yield broader benefits.
39	Halliday et al.	2017	Grit and Burnout in	Grit—the perseveranc	A survey of 548 UK	Higher grit correlated	Grit is a protective

			UK Doctors: A Cross-Sectional Study Across Specialties and Stages of Training	e and passion for long-term goals—may influence burnout in doctors. This study explores the relationship between grit and burnout across specialties and career stages in the UK.	doctors used the Short Grit Scale and Oldenburg Burnout Inventory. Participants included consultants, general practitioners (GPs), and trainees at various levels.	with lower burnout, particularly in consultants. GPs reported the highest burnout rates. Age and training stage partially explained grit differences, with consultants demonstrating the highest scores.	factor against burnout, particularly in experienced doctors. Resilience training focusing on grit could benefit younger doctors and GPs facing high burnout.
40	Wong et al.	2022	Impact of the COVID-19 Pandemic on the	The COVID-19 pandemic disrupted the training and well-	Surveys were administered to residents and program	A significant 84% of residents reported negative	The pandemic exacerbated burnout and

			Wellness of Canadian Plastic Surgery Residents	being of Canadian plastic surgery residents, adding stressors such as social isolation, redeployment, and reduced surgical opportunities to pre-existing issues like burnout and work-life imbalance.	directors across English-speaking Canadian plastic surgery programs. The study gathered quantitative and qualitative data on the impact of the pandemic on wellness and training.	impacts on their well-being, with 50% experiencing worsened emotional, social, and psychological wellness. Common stressors included uncertainty about future training and redeployment concerns. Internal support from programs was rated neutral or negative by 63% of residents.	disrupted surgical training. Programs should prioritize wellness strategies, such as better communication, improved resources, and support systems.
4	Zoorob	20	Insight	The early	A national	Burnout	Institutional

1	et al.	21	into Resident Burnout, Mental Wellness, and Coping Mechanisms Early in the COVID-19 Pandemic	phase of the COVID-19 pandemic heightened stress for residents across specialties, influencing burnout and coping mechanisms. This study sought to understand the impact of institutional support and mindfulness practices.	survey of 1,115 residents across 200 programs assessed well-being and resilience using validated tools, alongside demographic and coping strategy data.	prevalence was 51.5%, with 70% of residents distressed by pandemic-related emotional concerns. Residents using mindfulness practices like exercise and spending time with family reported slightly better wellness scores, though institutional support had	support significantly improves wellness, surpassing individual coping strategies. Enhancing programmatic support during crises can better address resident mental health.

						a stronger association with resilience.	
42	Suliman et al.	2024	Is Medical Training Solely to Blame? Generational Influences on the Mental Health of Medical Trainees	This study examines how generational traits, such as reliance on technology and social media overuse, interact with job demands to influence the mental health of Generation Y and Z medical trainees.	A survey of 326 trainees in Qatar analyzed factors like social media overuse, work-life balance, and job support in relation to burnout, stress, and resilience.	Social media overuse and poor work-life balance strongly correlated with stress and burnout among students and residents. Job support reduced burnout and increased resilience, whereas parenting style had no significant impact.	Generational characteristics, including technology reliance, contribute to trainee stress. Addressing these factors alongside improving job support could enhance mental health outcomes.
43	Ho and Kwek	2022	Levels of Burnout	Orthopaedic residents	A survey of 44 residents	Burnout prevalence	Addressing sleep

			and Its Association with Resilience and Coping Mechanisms Among Orthopaedic Surgery Residents: A Single Institution Experience from Singapore	face high burnout rates due to the demanding nature of their training. This study evaluates burnout prevalence and its association with resilience and coping mechanisms.	measured burnout using the Maslach Burnout Inventory and resilience with the Short Grit Scale. Coping strategies were assessed using the Brief COPE scale.	was 45.5%, with emotional exhaustion strongly linked to inadequate sleep and work-life conflict. Protective coping mechanisms included planning and positive reframing, while behavioral disengagement increased burnout risk.	issues and teaching effective coping strategies can reduce burnout in orthopaedic residents. Regular burnout screening is recommended for early intervention.
44	Rodríguez-Socarrás et al	2018	Lifestyle Among Urology Trainees	Urology trainees and young urologists	A survey of 412 urology trainees across 23	Most respondents had poor dietary	Lifestyle interventions focusing on diet,

			and Young Urologists in the Context of Burnout Syndrome	are prone to burnout, yet the role of lifestyle factors remains underexplored. This study investigates lifestyle habits, sleep patterns, and dietary intake in relation to burnout risk.	European countries collected data on lifestyle habits, sleep, and perceived health status.	habits, with 60% consuming less than one portion of fruit or vegetables daily. Sleep disturbances were common, with 60% reporting less than six hours of sleep per night. Burnout risk correlated with poor lifestyle satisfaction and sleep quality.	exercise, and sleep hygiene are critical for reducing burnout among urology trainees.
45	Abdelsattar et al.	2021	Lived Experiences of	The COVID-19 pandemic profoundly	Six 90-minute focus groups with	Key challenges included	The pandemic highlighted

			Surgical Residents During the COVID-19 Pandemic: A Qualitative Assessment	affected surgical residents' training, well-being, and professional identity. This study aimed to document the lived experiences of residents during the pandemic to inform future policies.	16 surgical residents from various institutions were conducted. Data were analyzed for themes related to personal, educational, and clinical impacts.	reduced surgical volume, disrupted education, uncertainty around personal safety, and emotional exhaustion. However, some residents reported increased camaraderie, flexibility in scheduling, and appreciation for leadership's efforts to provide support.	the need for robust institutional support systems that address both educational gaps and resident well-being during crises. Insights from this study can guide future disaster preparedness.
4	Koressel	20	Natural	Pediatric	A cohort of	Burnout,	Residency

6	et al.	20	History of Burnout, Stress, and Fatigue in a Pediatric Resident Cohort Over Three Years	residents face high burnout rates, but longitudinal data on its trajectory during training is limited. This study examined burnout, stress, fatigue, social connectedness, and resilience over three years.	33 pediatric residents was surveyed at six time points using tools like the Abbreviated Maslach Burnout Inventory, Perceived Stress Scale, and Connor-Davidson Resilience Scale. Data were analyzed using mixed-effects models.	stress, and fatigue peaked during the second year of residency and persisted throughout training. Resilience and social connectedness declined initially but showed some recovery by the end of residency.	training significantly impacts well-being, with mid-training being the most challenging period. Interventions to build resilience and maintain social connections are crucial for mitigating burnout
4 7	Shakir et al.	20 20	Relationship of Grit and Resilience	Neurosurgery residents experience high levels	A survey of 427 U.S. neurosurgery residents	Burnout prevalence was 33%, with	Grit and resilience protect against

			to Burnout Among U.S. Neurosurgery Residents	of burnout due to the intense nature of their training. This study investigated the relationship between grit, resilience, and burnout.	assessed burnout using the Maslach Burnout Inventory, grit using the Short Grit Scale, and resilience using the Connor-Davidson Resilience Scale.	emotional exhaustion and depersonalization being the primary contributors. Residents with high grit and resilience had lower burnout rates. Factors like fewer personal stressors, marriage, and children positively correlated with resilience and grit.	burnout in neurosurgery residents. Targeted interventions to strengthen these traits may improve well-being and reduce burnout.
48	Runyan et al.	2016	Impact of a Family	Burnout is prevalent	Twelve second-year	Residents reported	The wellness

			Medicine Resident Wellness Curriculum: A Feasibility Study	among family medicine residents, making wellness interventions critical. This study evaluated the feasibility and impact of a one-month wellness curriculum on stress, burnout, and empathy.	family medicine residents participated in weekly sessions focused on mindfulness, self-compassion, and gratitude. Pre- and post-intervention surveys measured burnout, empathy, stress, and self-compassion.	improvements in mindfulness and empathy, with reductions in stress and burnout indicators. However, the small sample size limited statistical power.	curriculum was well-received and showed promise in improving resident well-being. Expanding and refining the program could enhance its effectiveness.
49	Parks-Savage et al.	2018	Prevention of Medical Errors and Malpractice: Is	Physician burnout contributes to medical errors and	A review of literature on burnout, resilience, and medical	Resilience mitigates the effects of burnout, which is	Developing resilience through structured training in

			Creating Resilience in Physicians Part of the Answer?	malpractice. This article explores the role of resilience in reducing errors and enhancing patient safety.	errors was combined with recommendations for building resilience in medical education and practice.	linked to higher error rates. Programs that integrate self-care, mindfulness, and resilience training improve physician well-being and patient outcomes.	medical education and practice is essential for reducing errors and enhancing the overall healthcare system.
50	Blanchard et al.	2021	Resident Well-Being Before and During the COVID-19 Pandemic	This study investigates how the COVID-19 pandemic influenced burnout, resilience, and loneliness among	Surveys conducted between 2019 and 2020 at a single institution assessed burnout, resilience, and	Burnout rates remained stable before and during the pandemic, though frontline residents experienced	Stable well-being metrics during the pandemic suggest the potential effectiveness of preemptive institutional

				medical residents. Understanding these changes can inform strategies to support resident well-being during crises.	loneliness using validated scales. Data were collected from residents in multiple specialties pre- and post-pandemic onset.	higher burnout compared to non-frontline peers. Resilience and loneliness levels also stayed consistent, suggesting institutional measures may have buffered significant impacts.	responses. Future initiatives should focus on maintaining these supports during crises.
51	Aggarwal et al.	2017	Resident Wellness: An Intervention to Decrease Burnout and Increase	Burnout among residents negatively impacts well-being and patient care. This study	The curriculum included an initial 60-minute didactic session followed by weekly 15-	Of 272 invited residents, 69% attended the initial session. Participants reported	A structured wellness curriculum can improve resident resilience and

			Resiliency and Happiness	evaluates a 12-week wellness curriculum focusing on mindfulness, gratitude, and resilience-building exercises.	minute booster sessions. Participation spanned five residency specialties, with exercises peer-led by wellness champions.	increased mindfulness, optimism, and gratitude. Post-program, four departments voluntarily continued sessions, highlighting the program's acceptability and sustainability.	decrease burnout. Scaling such interventions across specialties may amplify benefits.
52	West et al.	2020	Resilience and Burnout Among Physicians and the General U.S.	Physician burnout is a pervasive issue, yet its association with resilience compared to	A cross-sectional national survey compared resilience (CD-RISC) and burnout	Physicians exhibited higher resilience scores than the general workforce. Resilience	Resilience training is beneficial but insufficient alone to combat burnout.

			Working Population	the general workforce remains unclear. This study examines resilience levels among U.S. physicians and their relationship to burnout.	(Maslach Burnout Inventory) in 5,445 physicians and 5,198 U.S. workers.	inversely correlated with burnout, but significant burnout persisted even among the most resilient physicians.	Systemic changes addressing workplace stressors are critical.
53	McFarland et al.	2017	Resilience of Internal Medicine House Staff and Its Association with Distress and Empathy in an Oncology Setting	Internal medicine residents face unique challenges in oncology settings, including exposure to patient deaths. This study explores the relationship	Fifty-six residents completed surveys assessing resilience (CD-RISC), distress (IES-R), and empathy before and after oncology rotations.	Higher resilience was associated with lower distress but not with empathy. While empathy decreased post-rotation, many	Resilience mitigates distress among oncology residents, emphasizing the need for resilience-building programs in such settings.

				between resilience, distress, and empathy in this context.	Data included death-related stress and coping strategies.	residents reported finding meaning in working with dying patients.	
54	Forbes et al.	2020	Resilience on the Run: An Evaluation of a Well-being Programme for Medical Interns	High psychological distress among junior doctors necessitates effective interventions. This study evaluates the "Resilience on the Run" program designed to build resilience and reduce burnout.	A prospective cohort study compared 24 interns undergoing the program to 29 controls. Participants completed psychological assessments at three intervals over three months.	While quantitative results were inconclusive due to small sample sizes, qualitative feedback indicated the program was well-received. Participants valued mindfulness training and practical stress managemen	Targeted resilience programs can enhance junior doctors' coping skills. Larger studies are needed to validate these findings.

						t strategies.	
55	Vasquez et al.	2021	Skills-Based Programs Used to Reduce Physician Burnout in Graduate Medical Education: A Systematic Review	Physician burnout is widespread in graduate medical education (GME). This systematic review examines skills-based programs designed to reduce burnout among GME trainees, identifying common elements and assessing their effectiveness.	The review followed PRISMA guidelines, analyzing 24 studies from 3534 abstracts that met eligibility criteria. Programs evaluated included mindfulness, stress management, resilience training, and wellness initiatives. Effectiveness was measured using validated	Eleven programs significantly reduced burnout, with most involving stress management, resilience training, and small group discussions. Programs during protected education time were most successful. However, outcomes varied widely, and high-quality randomized	Skills-based programs can reduce burnout but require standardization and rigorous evaluation. Future research should identify key components of successful interventions to inform program design.

					burnout scales.	controlled trials were scarce.	
56	Nituica et al.	2021	Specialty Differences in Resident Resilience and Burnout: A National Survey	Burnout is prevalent among resident physicians, but resilience may mitigate its effects. This study explores differences in resilience and burnout between medical and surgical residents across the U.S.	A cross-sectional survey of 682 residents used the Connor-Davidson Resilience Scale (CD-RISC) and Maslach Burnout Inventory. Demographics, institutional support, and family support were also analyzed.	Surgical residents reported higher resilience but similar burnout levels compared to medical residents. Resilience correlated positively with personal achievement and negatively with emotional exhaustion. Family and institutional	Resilience training and enhanced program support can reduce burnout, especially for high-risk groups. Tailored approaches addressing specialty-specific challenges are recommended.

						support were key protective factors.	
57	Peccoralo et al.	2022	Strategies for Enriching the Resident, Fellow, and Faculty Physician Experience: A System-Based Approach to Physician Well-Being	Physician burnout is a public health crisis. This study outlines a systems-level approach to addressing physician well-being through structured initiatives at the Mount Sinai Health System.	A three-pronged strategy included creating infrastructure, conducting needs assessments, and implementing targeted interventions based on survey results. Data were collected from faculty and residents through well-	Faculty burnout was 27.4%, while resident burnout was 54.8%. Interventions such as wellness day policies, enhanced mentorship, and reducing clerical burdens improved well-being metrics. Satisfaction with the Well-Being Champion	A system-based approach effectively addresses burnout and well-being. Continuous evaluation and iterative improvements are essential for sustaining positive outcomes.

					being surveys in 2018 and 2019.	program was 49.1% among residents.	
58	Simpkin et al.	2018	Stress From Uncertainty and Resilience Among Depressed and Burned Out Residents: A Cross-Sectional Study	Residents face high levels of burnout and depression, often linked to stress from uncertainty. This study investigates the relationship between stress from uncertainty, resilience, and mental health among pediatric residents.	Fifty residents across four pediatric residency programs completed surveys measuring stress from uncertainty, resilience (14-item Resilience Scale), depression, and burnout. Burnout was assessed using items from the Maslach Burnout	High stress from uncertainty negatively correlated with resilience (r = -0.60, p < 0.001). Residents with depression or burnout reported significantly higher uncertainty stress and lower resilience scores than their peers.	Enhancing resilience and tolerance for uncertainty could reduce depression and burnout in residents. Targeted training programs addressing these factors are recommended.

					Inventory.		
59	Sood et al.	2011	Stress Management and Resilience Training Among Department of Medicine Faculty: A Pilot Randomized Clinical Trial	Physician stress and burnout are significant concerns, yet few interventions effectively address them. This pilot study evaluates the Stress Management and Resiliency Training (SMART) program for faculty physicians.	Forty physicians were randomized into intervention and control groups. The SMART program included a single 90-minute session focusing on mindfulness, gratitude, and stress reduction. Outcomes were measured using validated scales for resilience,	Participants in the intervention group showed significant improvements in resilience (+9.8, p = 0.003), perceived stress (-5.4, p = 0.010), and anxiety (-11.8, p = 0.001) compared to controls. Overall quality of life also improved.	The SMART program is a feasible and effective intervention for reducing stress and enhancing resilience in physicians. Broader implementation and long-term evaluations are warranted.

					stress, and anxiety.		
60	Sood et al.	2014	Stress Management and Resiliency Training (SMART) Program Among Department of Radiology Faculty: A Pilot Randomized Clinical Trial	Physician stress, especially among radiologists, is increasing due to heavy workloads, diagnostic error concerns, and time constraints. This study evaluates the efficacy of the SMART program in reducing stress and enhancing resilience.	A randomized clinical trial with 26 radiologists divided into intervention and wait-list control groups. The intervention included a 90-minute SMART session focused on mindfulness and positive psychological frameworks, with follow-ups over 12 weeks. Outcomes	The intervention group showed significant reductions in perceived stress and anxiety and improvements in mindfulness and quality of life. However, resilience improvements were not statistically significant compared to the control group.	The SMART program is a feasible, effective intervention for reducing stress and improving mindfulness and quality of life in radiologists. Further studies with larger sample sizes are recommended.

					were measured using stress, anxiety, mindfulness, and resilience scales.		
61	Riese et al.	2024	The Effects of a Resiliency Intervention Program on Indicators of Resiliency and Burnout in Psychiatry Residents	Psychiatry residents experience high levels of burnout due to emotional and psychological demands. This study evaluates a mindfulness-based resiliency program.	A six-session intervention was conducted with 27 psychiatry residents, focusing on mindfulness, stress management, and self-regulation. Pre- and post-program assessments measured	Residents experiencing burnout showed significant reductions in perceived stress and emotional exhaustion, along with increased mindfulness and resilience. Those not meeting burnout criteria	Mindfulness-based interventions are effective in reducing burnout and increasing resilience in psychiatry residents. Mindfulness training should be a core component of wellness

					burnout, mindfulness, and resilience.	showed no significant changes.	programs.
62	Orr et al.	2019	The Fostering Resilience Through Art in Medical Education (FRAME) Workshop: A Partnership with the Philadelphia Museum of Art	Burnout among internal medicine residents is prevalent, and exposure to medical humanities may foster resilience. This study evaluates an art-based intervention to reduce burnout.	A single, 4-hour workshop combining artistic analysis and reflection was conducted with 17 internal medicine residents. Burnout was assessed pre- and post-workshop using the Maslach Burnout Inventory.	Emotional exhaustion and depersonalization scores showed moderate reductions post-workshop. Participants reported enhanced reflection, perspective-taking, and community building.	The FRAME workshop shows promise as a novel intervention to reduce burnout. Further studies are needed to optimize its structure and assess its long-term impact.
6	Dam et	20	The	Burnout is	A cross-	Residents	Grit is a

3	al.	19	Relationship Between Grit, Burnout, and Well-being in Emergency Medicine Residents	prevalent in emergency medicine residents, and grit may be a protective factor. This study examines the relationship between grit, burnout, and well-being.	sectional survey of 222 emergency medicine residents assessed burnout (Maslach Burnout Inventory), grit (Short Grit Scale), and well-being (WHO-5 Index)	with high grit had significantly lower rates of burnout and poor well-being. Grit positively correlated with personal accomplishment and negatively with emotional exhaustion and depersonalization.	protective factor against burnout and low well-being. Interventions to cultivate grit may improve resident resilience and mental health.
64	Du et al.	2024	The Relationship Between Self-Efficacy,	Pediatric residents in China face high burnout rates. This study	A survey of 190 pediatric residents measured burnout (Physicians'	Higher self-efficacy was associated with lower burnout through	Resilience mediates the relationship between self-efficacy

			Resilience, and Job Burnout in Pediatric Residents: A Cross-Sectional Study in Western China	explores the mediating role of resilience in the relationship between self-efficacy and burnout.	Career Burnout Questionnaire), self-efficacy, and resilience. Mediation analysis examined resilience's role in mitigating burnout.	increased resilience. Female residents reported lower self-efficacy and higher burnout compared to males.	and burnout. Enhancing resilience through targeted interventions may reduce burnout among pediatric residents.
65	Hopkins et al.	2024	Trainee Growth vs. Fixed Mindset in Clinical Learning Environments: Enhancing, Hindering, and Goldilocks Factors	This study explores how clinical learning environments influence trainees' adoption of growth or fixed mindsets. A growth mindset is associated	Sixteen oncology and medical education trainees participated in a tabletop simulation game called "Mindset" followed by facilitated debriefings. Discussions	Internal factors like passion and grit promoted growth mindsets but could hinder development when excessive (Goldilocks principle). External	The balance of internal and external factors significantly influences mindset adoption. Educational strategies should foster a supportive

				with resilience and better coping strategies, while a fixed mindset is linked to burnout and decreased learning.	focused on factors promoting growth or fixed mindsets, analyzed using template analysis.	factors, including role modeling and feedback, supported growth mindsets, while competition and burnout promoted fixed mindsets.	learning environment and balance stressors to encourage growth mindsets among trainees.
66	Ng et al.	2018	Unpacking the Literature on Stress and Resiliency: A Narrative Review Focused on Learners	The operating room (OR) is a high-stress environment that challenges surgical trainees' performance and learning.	A narrative review synthesized literature from fields including medical education, surgery, and psychology. Themes included	Common OR stressors include fatigue, interpersonal conflicts, and time pressure. Effective coping strategies	Resilience training and stress management techniques are essential for improving surgical trainees'

			in the Operating Room	This review examines stress, coping mechanisms, and resilience in the OR context.	stressors, coping strategies, and resilience's role in mitigating stress.	include mental rehearsal and mindfulness. Resilience emerged as a critical factor in reducing stress and improving performance.	performance and well-being. Future research should focus on developing targeted interventions to enhance resilience in high-pressure environments.
67	Odom et al.	2022	Using Photovoice to Explore Family Medicine Residents' Burnout Experiences and	While burnout in medical residents is well-documented, qualitative insights into their experiences	Family medicine residents took photographs representing burnout and resilience. These images and	Burnout themes included physical exhaustion, self-neglect, and being overwhelmed. Resilience strategies	Photovoice provides unique insights into burnout and resilience. Residency programs

			Resiliency Strategies	and resilience strategies are limited. This study uses Photovoice, a participatory method, to explore these perspectives.	accompanying captions were discussed in small groups, with data analyzed using a hermeneutic phenomenology approach.	involved self-care, nurturing relationships, and seeking nature. Burnout images were dull and monochromatic, while resilience images often depicted vibrant, natural settings.	can use this methodology to engage residents in wellness discussions and identify effective resilience-building strategies.
68	Reed et al.	2018	Variability of Burnout and Stress Measures in Pediatric Residents: An Explorator	Pediatric residency is associated with high burnout rates. This study explores short-term	Surveys assessing burnout, stress, resilience, and mindfulness were administered	Emotional exhaustion and depersonalization increased over time, while empathy	Longitudinal interventions targeting mindfulness and resilience could mitigate

			y Single-Center Study from the Pediatric Resident Burnout–Resilience Study Consortium	stability in burnout and related factors and their predictive power for wellness outcomes.	to 108 pediatric residents at two time points, three months apart. Cross-sectional and longitudinal analyses were conducted.	decreased. Resilience and mindfulness were protective against burnout. Mindfulness and self-compassion emerged as critical predictors of emotional exhaustion reduction.	burnout and enhance well-being in pediatric residents. Regular monitoring of these factors is recommended to guide interventions.
69	Ricker et al.	2021	Well-Being in Residency: Impact of an Online Physician Well-Being Course on Resiliency	Incoming residents often experience high stress and burnout. This study evaluates the Andrew Weil Center	Eighty-seven incoming residents from 15 specialties completed the course, which included modules on	Emotional exhaustion and depersonalization significantly decreased post-course, while resilience	Online well-being courses effectively reduce burnout and enhance resilience among new

			and Burnout in Incoming Residents	for Integrative Medicine's online course on well-being and its impact on resilience and burnout.	mindfulness, resilience, and stress management. Pre- and post-course assessments measured burnout, resilience, and gratitude.	improved. Gratitude remained unchanged. Residents incorporated wellness behaviors such as improved sleep and exercise into their routines.	residents. Scaling such initiatives could benefit broader groups of trainees.
